# Geographic variation and plasticity in climate stress resistance among southern African populations of *Ceratitis capitata* (Wiedemann) (Diptera: Tephritidae)

**DOI:** 10.1038/s41598-018-28259-3

**Published:** 2018-06-29

**Authors:** Christopher W. Weldon, Casper Nyamukondiwa, Minette Karsten, Steven L. Chown, John S. Terblanche

**Affiliations:** 1 Flies of Economic Importance Research Group, Department of Zoology and Entomology, University of Pretoria, Private Bag X20, Hatfield, 0028 South Africa; 2 DST-NRF Centre for Invasion Biology, Department of Conservation Ecology and Entomology, Stellenbosch University, Private Bag X1, Matieland, 7602 South Africa; 3 School of Biological Sciences, Monash University, Victoria, 3800 Australia; 4 Present Address: Department of Biological Sciences and Biotechnology, Botswana International University of Science and Technology (BIUST), Private Bag 16, Palapye, Botswana

## Abstract

Traits of thermal sensitivity or performance are typically the focus of species distribution modelling. Among-population trait variation, trait plasticity, population connectedness and the possible climatic covariation thereof are seldom accounted for. Here, we examine multiple climate stress resistance traits, and the plasticity thereof, for a globally invasive agricultural pest insect, the Mediterranean fruit fly, *Ceratitis capitata* (Wiedemann) (Diptera: Tephritidae). We also accounted for body size and population genetic connectivity among distinct populations from diverse bioclimatic regions across southern Africa. Desiccation resistance, starvation resistance, and critical thermal minimum (CT_min_) and maximum (CT_max_) of *C. capitata* varied between populations. For thermal tolerance traits, patterns of flexibility in response to thermal acclimation were suggestive of beneficial acclimation, but this was not the case for desiccation or starvation resistance. Population differences in measured traits were larger than those associated with acclimation, even though gene flow was high. Desiccation resistance was weakly but positively affected by growing degree-days. There was also a weak positive relationship between CT_min_ and temperature seasonality, but CT_max_ was weakly but negatively affected by the same bioclimatic variable. Our results suggest that the invasive potential of *C. capitata* may be supported by adaptation of tolerance traits to local bioclimatic conditions.

## Introduction

While it is widely accepted that the geographic distributions of animal and plant species can be tightly correlated with environmental factors, the evolutionary and ecological determinants of such correlations are less clear. Consequently, variation in geographic ranges and the temporal dynamics thereof cannot yet be predicted with great confidence or precision for the vast majority of terrestrial biodiversity^1^. Apart from genetic factors such as inbreeding depression or low additive genetic variance that may explain trait variation and adaptive evolutionary potential in small populations^2,3^, an emerging body of research proposes that ecological traits dictate species-environment relationships through fundamental evolutionary limits at the species level^4–6^. The generality of such an explanation requires further scrutiny because it presupposes limited intraspecific variation. This is problematic because empirical evidence for the role of intraspecific variation in tolerance of environmental factors is not comprehensive for most taxonomic groups, and is even more depauperate for insects^7^. Modelling that incorporates adaptive capacity suggests that some species of Australian *Drosophila* are less vulnerable to climate change than previously thought^8^. But direct comparison of *Drosophila* species with overlapping geographic distributions show different evolutionary responses to climatic change^9^. Improving understanding of intraspecific variation in insect tolerance of climate stress is not only of fundamental importance, but also of applied relevance.

Biological invasions serve as excellent models to test hypotheses about evolutionary mechanisms setting species range limits. The characteristics of invasive species vary between taxonomic groups, but some notable features are common^10^. For terrestrial arthropods, invasive species are more likely to be associated with human disturbance^11^. They tend to have *r*-selected life history strategies that include high intrinsic growth rates, small body size, fast time to maturity, and multivoltinism^10^, and exhibit physiological tolerance, or greater plasticity of tolerance, to suboptimal conditions^11–13^. Invasive terrestrial arthropods also tend to be well adapted for dispersal^10^. Further, they are likely to have a genetic architecture (e.g., high additive genetic variance, epistasis) capable of more easily or rapidly evolving in response to natural selection in a novel environment^2,14,15^. However, what is apparent from modelling of invasions is that even in situations where propagule pressure of invading species is high, the suitability of the novel environment for a species is an overriding factor in its establishment^13,16–18^. Unravelling the factors associated with successful invasion provide a basis for mitigating the introduction, establishment or spread of potentially invasive species, and may also inform the implementation of suitable control measures^19^.

The suitability of novel environments for invasive species may relate to either or both the baseline tolerance of environmental extremes resulting from local adaptation, or the level of flexibility (or plasticity) in tolerance traits. In a recent comparison of invasive insects and closely-related non-invasive species, the invasive species were characterised by elevated lower developmental temperatures but lower sums of effective temperature, which would favour the invasive species when introduced to thermally suitable environments^20^. Furthermore, in the invasive insect *Coruthucha siliata*, the intrinsic rate of increase was enhanced by increases in both mean and extreme high temperatures^21^. Phenotypic plasticity of environmental tolerance traits has been proposed as a property that increases the likelihood of a species becoming invasive^10,12^. This hypothesis is supported by some invertebrate systems. In the Swiss Alps, for example, an invasive slug, *Arion lusitanicus* (Mabille), was able to maintain high levels of fitness and had a more adaptive phenotypic plasticity compared with a congeneric native slug, *A. fuscus* (Mueller) enabling it to invade high altitudes and survive climate warming^22^. On sub-Antarctic Marion Island, the form of plasticity exhibited by an invasive springtail species is such that they have an advantage over indigenous species under the drying conditions predicted from a changing climate^12^.

The Mediterranean fruit fly, *Ceratitis capitata* (Wiedemann) (Diptera: Tephritidae), is recognised as a highly invasive insect pest^23,24^. Historical records of its spread and analyses of biochemical and molecular markers^25,26^ suggest that the species originated in central eastern Africa and has a native range throughout sub-Saharan Africa. In association with the growth and development of the international trade of fresh fruit^23,24^, *C. capitata* has been introduced, established successfully, and expanded its range throughout many tropical, subtropical, and mild temperate habitats of the world^27^. Its now almost pandemic distribution in suitable climates (excluding only central and eastern Asia) and highly polyphagous use of fruit hosts (reared from over 150 plant species in Africa alone^28^) has led to this species being considered the most economically damaging pest of horticulture in the world. Economic damage is incurred from direct crop losses, pre- and post-harvest control costs, and limited or loss of access to fly-free export markets^29^. The ecological impact of *C. capitata* in its invasive range has been far more modest; in combination with two other invasive fruit fly species, *Bactrocera zonata* (Saunders) and *C. rosa* Karsch, *C. capitata* competitively excludes an indigenous fruit fly, *C. catoirii* Guérin-Mèneville, on the island of Réunion^30^.

If the rapid global colonisation of *C. capitata* is to be fully understood, its plastic responses to environmental stresses need to be known in addition to baseline responses to constant (stable) environments. Within-generation changes in the thermal tolerance of *C. capitata* have been reported in relation to prior thermal environment under both laboratory^31–34^ and semi-natural conditions^35^. Acclimation and acclimatisation occur rapidly^34^, with changes in thermal tolerance tracking daily temperature fluctuations^35^. In one study^33^, thermal performance breadth for muscular function was approximately 6.1 °C–42.4 °C when *C. capitata* were acclimated at 25 °C. The thermal performance breadth increased by a small amount when flies were acclimated to 20 °C, but declined when flies were acclimated to 30 °C, because tolerance of stressful low temperatures changes to a greater extent than tolerance of stressful high temperatures^33^. Knowledge of the range of temperatures over which insect activity is possible is fundamental for the mechanistic determination of range limits and habitat suitability^36^, and has been used successfully to predict the population persistence of African tephritids^35^. Similarly, physiologically-based models of habitat suitability require knowledge of the water and nutrient requirements and reserves of individuals for survival. While nutrient intake targets have received some attention^37–40^, water relations and starvation resistance of tephritid fruit flies, in general, are far less well studied than their thermal relations, and the flexibility in these former traits even less so. The pupal stage of *C. capitata* is quite tolerant to low soil moisture conditions, with approximately 60% survival when held at 50% relative humidity, but is susceptible to drowning when immersed in water for over one hour, which may be anticipated in water-logged soils following heavy rain^30^. In contrast, adult *C. capitata* are more resistant to desiccation relative to other *Ceratitis* species, and the same pattern is evident for starvation resistance^41^. To date, there have been no studies published on the way in which desiccation resistance of adult *C. capitata* varies due to prior environmental experience.

The aim of this study was to examine population-level variability and phenotypic plasticity for multiple climate stress resistance traits in a globally invasive agricultural pest insect, the Mediterranean fruit fly, *C. capitata*. We determined the desiccation and starvation resistance, lower and upper critical thermal limits, and the plasticity thereof, while accounting for life-history variation (e.g. body size), among populations from different bioclimatic regions across sub-Saharan Africa. Gene flow alters the potential for local adaptation by increasing genetic variation. While this can reduce the potential for local adaptation under certain conditions^42^, it also provides variation on which natural selection can act to remove deleterious alleles from a population^43^. However, few studies account for population relatedness and whether it is a significant covariate explaining variation in stress resistance traits. For this reason, we went on to establish whether patterns of climate stress resistance in populations of *C. capitata* in southern Africa were associated with bioclimatic characteristics and the evolutionary relationships among sampled populations.

## Results

### Initial condition

**Figure 1:**
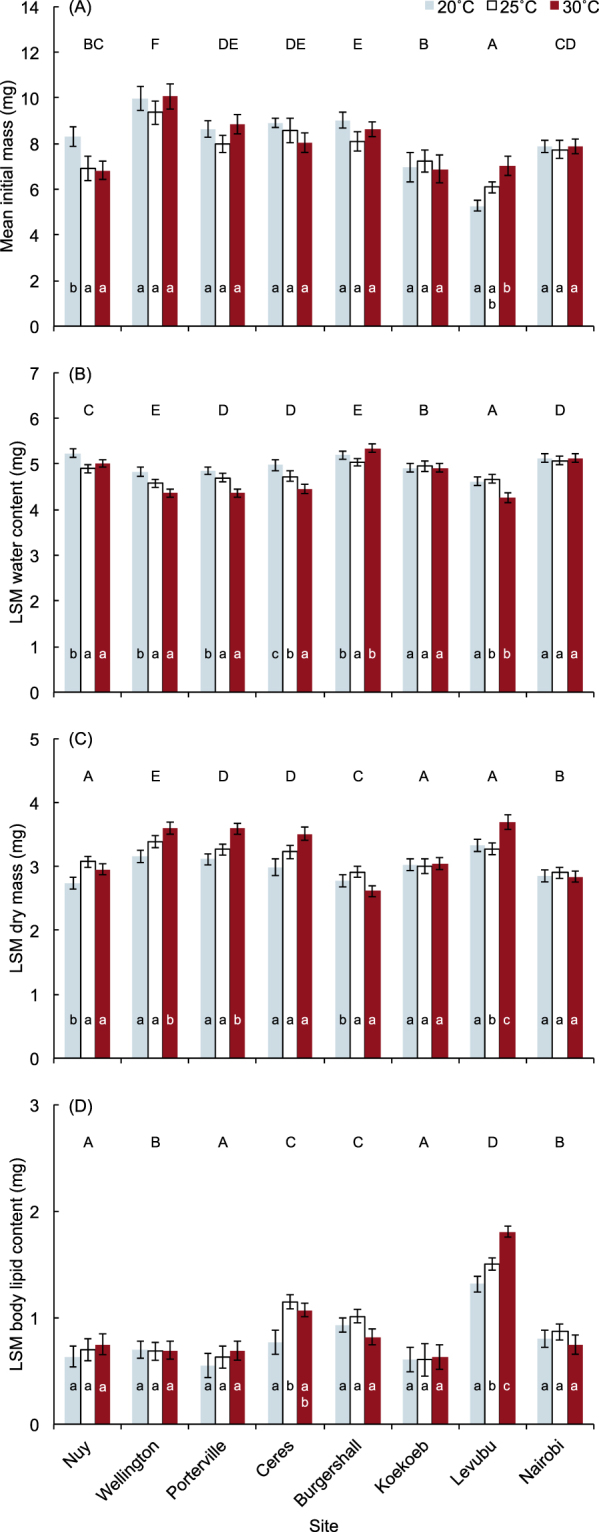
Mean initial mass (**A**) and least squares means (LSM) body water content (**B**), dry mass (**C**) and lipid content (**D**) of adult *Ceratitis capitata* from different sites in sub-Saharan Africa and acclimated at three temperatures. LSM values were generated using a generalised linear model, with initial body mass at its mean (i.e., 7.96 mg for this cohort of flies) to account for effects of body size. Error bars represent ±1 SE. Sites labeled with the same capital letter are not significantly different from each other, and bars overlaid with the same lower case letter indicate acclimation temperatures not significantly different from each other within a site (least significant difference tests: p > 0.05).

*Ceratitis capitata* populations were sampled at eight sites in regions of southern Africa with different climates spanning a latitudinal range of *c*. 32° (Table 1). After adults from each population were acclimated to one of three temperatures (20, 25 and 30 °C), body mass, water content, dry mass, and lipid content was measured before they were subjected to stress tolerance assays. Initial body mass was significantly different among sites, but the effect of site had a relatively small effect on initial body mass (Table S1). Flies from Wellington (mean ± 1 s. e. = 9.8 ± 0.3) were significantly heavier than those from all other sites (indicated by model parameter estimates), and flies from Levubu (6.0 ± 0.2) had the lowest initial body mass (Fig. 1A). The effect of acclimation nested within locality had no significant effect on initial body mass.

**Table 1:** Location and bioclimatic characteristics of sites sampled for *C. capitata*. Bioclimatic characteristics of each site are extracted from Metzger *et al*.^44^.

Site	Coordinates	PET (sd)	GDD	Aridity index	Temp (sd)	GEnS
Nuy	33.67°S 19.62°E	5406.975013	6088.1	2731	4010	84
Wellington	33.39°S 18.57°E	4917.963363	6292.5	3069	3470	84
Porterville	33.01°S 19.00°E	5797.877093	5734.4	3107	4337	89
Ceres	33.34°S 19.57°E	5116.432232	4764.2	3003	4132	70
Burgershall	25.06°S 31.05°E	3237.095593	7357.0	6570	2685	94
Koekoeb	28.47°S 20.46°E	6027.286439	7302.7	852	5218	96
Levubu	23.05°S 30.17°E	2955.874992	7108.5	6298	2845	94
Nairobi	1.28°S 36.82°E	1487.523873	6694.6	5444	1195	88

PET (sd): potential evapotranspiration seasonality (standard deviation of monthly mean potential evaporation × 100).

GDD: growing degree-days with 0 °C base (calculated on monthly temperature means above 0 °C × number of days in the month).

Aridity index: mean annual precipitation/mean annual evapotranspiration; sites with the lowest values are the most arid.

Temp (sd): temperature seasonality (standard deviation of monthly mean temperature × 100).

GEnS: Stratification number allocated in Metzger *et al*.^44^.

Initial body water and dry mass were significantly affected by site, acclimation within site, and initial body mass (Table S1). Initial body mass accounted for the largest component of the variance in initial body water and initial dry mass, although for both, the variance accounted for by the effect of site was approximately three times greater than that accounted for by acclimation. Both initial body water and dry mass were positively related to initial body mass (Table S2). Overall, initial body water comprised 61.3 ± 0.3% of initial body mass (with dry mass comprising the remainder), and in general, sites with higher initial body water (Fig. 1B) tended to have lower initial dry mass (Fig. 1C). When controlling for the strong effect of initial body mass, flies from Levubu had the lowest initial body water, and those from Burgershall and Wellington had the highest initial body water (Fig. 1B). In general, flies acclimated at 20 °C had higher initial body water than those acclimated at 30 °C, but this pattern was not evident in flies from Nairobi, Burgershall or Koekoeb, where acclimation appeared to have little effect on initial body water (Fig. 1B).

Initial body lipid content, overall, comprised 28.1 ± 0.7% of initial dry mass. Again, site, acclimation within site, and initial mass significantly affected initial body lipid content, with initial mass accounting for the largest component of the variance (Table S1). The variance accounted for by site was far greater than that accounted for by acclimation. Flies from Koekoeb, Nuy and Porterville had the lowest initial body lipid content, whereas those from Levubu had the highest initial body lipid content (Fig. 1D). Within sites, there was a general trend for flies acclimated at 25 °C to have higher initial body lipid content than those acclimated at 20 °C, but lipid content of those acclimated at 30 °C exhibited no consistent pattern.

### Desiccation and starvation resistance, and thermal tolerance traits

**Figure 2:**
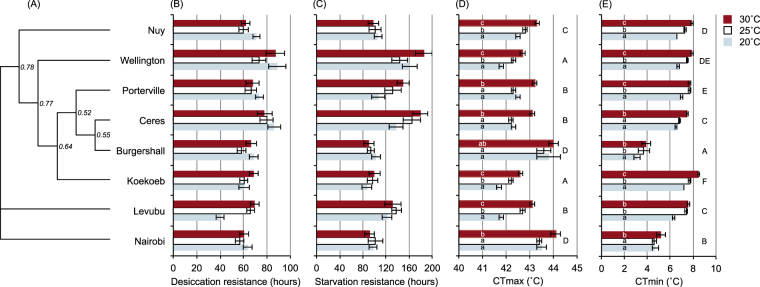
Desiccation and starvation resistance, lower and upper critical thermal limits, and the plasticity thereof when acclimated at three temperatures, in *Ceratitis capitata* sampled from eight sites in southern Africa. (**A**) Unrooted neighbour-joining tree for the eight populations based on Nei’s genetic distance. The number at each node indicates the bootstrap values after 10000 bootstrap replicates. Only bootstrap values above 50% are shown. (**B**) Mean survival time during a desiccation resistance assay. (**C**) Mean survival time during a starvation resistance assay. (**D**) Critical thermal maximum (CT_max)_. (**E**) Critical thermal minimum (CT_min_). Error bars represent ±1 SE. For CT_max_ and CT_min_, sites labeled with the same capital letter are not significantly different from each other, and bars overlaid with the same lower case letter indicate acclimation responses not significantly different from each other within a site (least significant difference tests: p > 0.05).

Time-to-death stress assays were used to determine the desiccation and starvation resistance, dehydration tolerance and lipid metabolism of adult *C. capitata* from each population and acclimation treatment. The minimal adequate model describing survival time of *C. capitata* during a desiccation resistance assay included site (χ^2^ = 85.36, df = 7, P < 0.001) and acclimation nested within site (χ^2^ = 44.11, df = 16, P < 0.001). Body mass did not significantly affect desiccation resistance and was removed from the final model. Overall mean desiccation resistance (±1 s.e.) was highest in flies from Wellington (83.1 ± 4.1 hours) and Ceres (81.4 ± 3.4 hours); survival of these was significantly longer than the reference category (Burgershall: 64.7 ± 2.3 hours). Flies from Levubu (58.6 ± 2.6 hours) exhibited the lowest recorded desiccation resistance, and their survival time was significantly lower than the reference category. Overall mean desiccation resistance of other sites was not significantly different from the reference category. Within sites, acclimation did not consistently affect desiccation resistance (Fig. 2B). The effects of acclimation were small (mean acclimation response difference = 1.0 ± 4.5 hours) relative to differences between sites except for flies from Levubu, where acclimation at 25 and 30 °C led to an over 50% increase in desiccation resistance.

During a starvation resistance assay, mortality risk of *C. capitata* was best described by a model including site (χ^2^ = 120.70, df = 7, P < 0.001) and initial mass as a covariate, although the effect of initial mass was not significant (χ^2^ = 3.39, df = 1, P = 0.066). Acclimation nested within site had no significant effect on starvation resistance and was dropped from the final model (mean acclimation response difference = 13.1 ± 7.8 hours). Flies from Burgershall (which represented the reference category) had the lowest starvation resistance (106.2 ± 0.4 hours). Starvation resistance was highest in flies from Wellington (182.2 ± 0.9 hours) and Ceres (178.4 ± 0.6 hours), then Porterville (147.7 ± 0.6 hours) and Levubu (144.2 ± 0.4 hours; Fig. 2C), all of which survived significantly longer than the reference category. There was no significant difference in starvation resistance of the remaining sites and the reference category.

**Figure 3:**
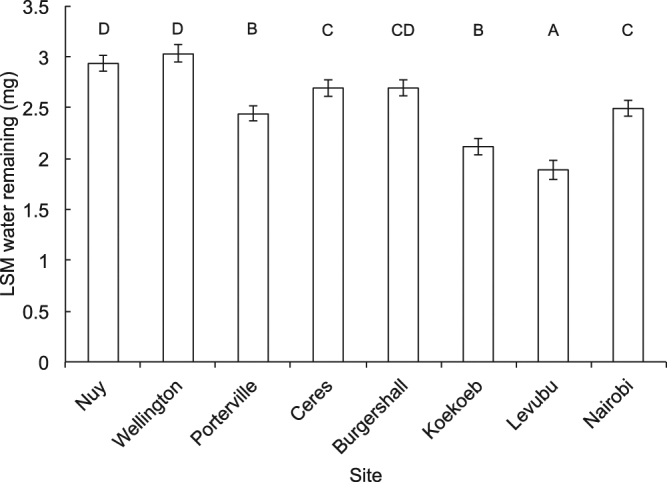
Least squares means (LSM) water remaining at death of adult *Ceratitis capitata* from different sites in sub-Saharan Africa that were acclimated at three temperatures and subjected to a desiccation resistance assay. LSM values were generated using a generalised linear model, with estimated total body water at its mean (4.69 mg) to account for water initially available. Error bars represent ±1 SE. Sites labeled with the same capital letter are not significantly different from each other (least significant difference tests: p > 0.05).

Dehydration tolerance (measured as body water remaining at death) of *C. capitata* subjected to the desiccation resistance assay was significantly affected by site and initial body mass (Table S3). Flies from Burgershall, Nuy and Wellington had the lowest dehydration tolerance (i.e., they had the highest body water at death, taking into account their initial body mass; Fig. 3). Dehydration tolerance decreased significantly as initial body mass increased (parameter estimate: 0.081 ± 0.019). There was no statistical evidence for acclimation nested within site affecting dehydration tolerance.

**Figure 4:**
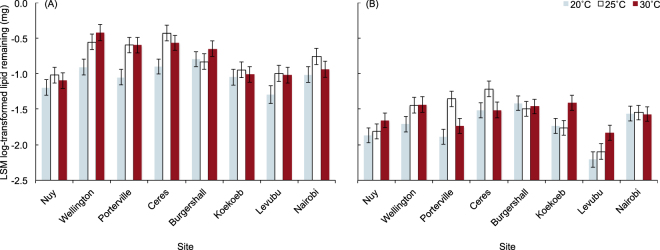
Least squares means (LSM) lipid remaining in adult *Ceratitis capitata* after being subjected to (**A**) desiccation and (**B**) starvation resistance assays. LSM values were calculated using a generalised linear model, with initial mass and survival time at their means (initial mass = 7.71 mg; survival time = 94.79 hours).

Lipid metabolism during desiccation and starvation, expressed as lipid remaining at death, was significantly affected by site, acclimation nested within site, and assay nested within acclimation (Table S4). Flies from Levubu and Nuy had the lowest levels of lipid remaining at death, whereas Burgershall, Ceres and Wellington had the highest (Fig. 4). Acclimation only had an effect within populations from Levubu and Porterville, but the pattern was not consistent. More lipids remained after desiccation resistance assays (Fig. 4A) than after starvation resistance assays (Fig. 4B). This was likely associated with the significant effect of survival time, where flies that survived the longest had the most depleted lipid levels (parameter estimate: −0.002 ± 0.000). There was no effect of initial body mass on survival, suggesting that lipid reserves of larger flies are not contributing to differences within the species. Assay explained the most variance of the manipulated factors, followed by site, and then acclimation and survival time (Table S4).

To assess the thermal tolerance of adult *C. capitata* from each population and acclimation treatment, their critical thermal maximum (CT_max_) and minimum (CT_min_) were recorded. CT_max_ was significantly affected by site, and acclimation within site, but they contributed very little to the variance explained by the model (Table 2). The populations with the highest CT_max_ were Burgershall and Nairobi, followed by Nuy (Fig. 2D). CT_max_ was lowest in flies from Koekoeb and Wellington. Within sites, flies acclimated at 30 °C tended to have a CT_max_ significantly higher than those acclimated at 20 °C, with a mean acclimation response difference of 0.76 ± 0.11 °C. CT_min_ was also significantly affected by site, and acclimation within site. In this case, site accounted for a higher percentage of the model variance than acclimation (Table 2). CT_min_ was highest in flies from Koekoeb, then Porterville, and lowest in flies from Burgershall (Fig. 2E). Similar to CT_max_, CT_min_ of flies acclimated at 30 °C tended to be higher than those acclimated at 20 °C, with a mean acclimation response difference of 1.01 ± 0.11 °C.

**Table 2:** Summary of the linear models that describe the effects of acclimation nested within site on CT_max_ and CT_min_ in *C. capitata*.

Effect	SS	df	MS	*F*	*P*	% variance
CT_max_						
Intercept	698796.7	1	698796.7	3122208	<0.001	>99.9
Site	97.1	7	13.9	62	**<0.001**	<0.1
Acclimation	63.0	16	3.9	18	**<0.001**	<0.1
Error	163.18	395	0.36			<0.1
CT_min_						
Intercept	16286.3	1	16286.3	62304	<0.001	95.7
Site	540.4	7	77.2	295	**<0.001**	3.2
Acclimation	92.1	16	5.8	22	**<0.001**	0.5
Error	103.3	395	0.3			0.6

### Association with bioclimatic variables and genetic relatedness

The association of climate stress resistance in populations of *C. capitata* in southern Africa with bioclimatic characteristics and the evolutionary relationships among sampled populations was evaluated using a two-step procedure. Mean values for desiccation and starvation resistance, lipid content, and CT_max_ and CT_min_, as well as the acclimation response difference for each of these variables, were related to all bioclimatic characteristics using ordinary least-squares regression. The minimal adequate model for each of these analyses was then extended to phylogenetic least squares regression. Bioclimatic characteristics assessed were those identified by Metzger, *et al*.^44^ as the most important for constructing their global environmental stratification: potential evapotranspiration seasonality [PET (sd)], growing degree-days with 0 °C base (GDD), aridity index, and temperature seasonality [Temp (sd)] (see Materials and Methods for definitions). Desiccation resistance of *C. capitata* sampled from each site was significantly negatively correlated with GDD (Table 3). There was also a marginally significant effect of GDD on starvation resistance, with starvation resistance decreasing with GDD. All other bioclimatic variables were removed from the minimal adequate ordinary least-squares regression models for desiccation and starvation resistance. Phylogenetic least-squares regression only marginally improved the fit of models for desiccation and starvation resistance, but with phylogeny not being correlated with either measured trait.

**Table 3:** Coefficients from the minimal adequate ordinary least-squares regression (OLS) and phylogenetic generalised least squares regression (PGLS) models for mean desiccation resistance, starvation resistance, lipid content, critical thermal minimum (CT_min_) and critical thermal maximum (CT_max_) of *C. capitata* populations.

Effect	OLS					PGLS					
	Estimate	S.E.	*t*	*P*	AIC	Estimate	S.E.	*t*	*P*	AIC	*λ*
Desiccation											
(Intercept)	107.676	16.215	6.641	0.001	54.801	107.397	16.451	6.528	0.001	52.993	0.000
GDD	−0.007	0.003	−2.647	**0.038**		−0.007	0.003	−2.584	**0.042**		
Starvation											
(Intercept)	255.376	56.027	4.558	0.004	74.639	252.962	56.969	4.440	0.004	72.866	0.000
GDD	−0.021	0.009	−2.411	0.052		−0.020	0.009	−2.317	0.060		
Lipid content											
(Intercept)	1.114	0.848	1.314	0.280	−1.026	1.095	0.867	1.262	0.296	−2.873	0.000
PET	−0.000	0.000	−1.121	0.344		−0.000	0.000	−1.112	0.347		
GDD	−0.000	0.000	−1.530	0.224		−0.000	0.000	−1.498	0.231		
Aridity	0.000	0.000	1.828	0.165		0.000	0.000	1.814	0.167		
Temp (sd)	0.000	0.000	1.411	0.253		0.000	0.000	1.418	0.251		
CT_min_											
(Intercept)	3.556	1.208	2.943	0.026	27.586	3.701	1.162	3.184	0.019	25.015	0.000
Temp (sd)	0.001	0.000	2.669	**0.037**		0.001	0.000	2.696	**0.036**		
CT_max_											
(Intercept)	42.573	1.070	39.778	< 0.001	8.766	42.626	1.050	40.581	< 0.001	6.528	0.000
GDD	0.000	0.000	1.256	0.264		0.000	0.000	1.189	0.288		
Temp (sd)	−0.000	0.000	−2.927	**0.033**		−0.000	0.000	−2.962	**0.031**		

The minimal adequate least squares regression model was determined using step-wise deletion of least significant terms based on improvement of Akaike’s information criterion (AIC). Predictor variables in the full model were potential evapotranspiration seasonality [PET (sd)], growing degree-days with 0 °C base (GDD), aridity index, and temperature seasonality [Temp (sd)]. Phylogenetic correlation (on a scale of 0 to 1) is given by *λ*.

Both CT_min_ and CT_max_ of *C. capitata* from sampled sites were affected by Temp (sd). In the case of CT_min_, mean values increased slightly with Temp (sd). All other bioclimatic variables were removed from the ordinary least-squares minimal adequate model. In contrast, CT_max_ decreased slightly as Temp (sd) increased. GDD was retained in the minimal adequate model for CT_max_. Phylogenetic least-squares regression only marginally improved the fit of models for CT_min_ and CT_max_. These traits were not correlated with phylogeny.

The acclimation response differences for desiccation resistance, starvation resistance, CT_max_ and CT_min_ of flies from each site were not significantly associated with any of the tested bioclimatic variables (Table S5), or with the among-population phylogeny.

## Discussion

Desiccation resistance, starvation resistance and thermal tolerance of *C. capitata* varied geographically between populations within sub-Saharan Africa. This parallels results obtained for life history and behavioural traits of *C. capitata* over its native and invasive range^24,45–47^. Furthermore, it is also in keeping with results from other studies that have demonstrated geographic variation in the water balance, nutritional or thermal traits of insects and other arthropods^48–55^. In *D. melanogaster*, for example, differences have been detected in desiccation resistance, starvation resistance, and size between geographic regions (i.e., populations from northern and southern Australia), although there was higher variability between strains collected at multiple sites in each region^50^. These results, and those found in *C. capitata*, likely indicate adaptation in stress tolerance traits facilitate survival of populations in the environmental conditions that prevail where they were collected. The role of local adaptation to environmental conditions in shaping tolerance traits is strengthened by the absence of a phylogenetic signal in population means or plasticity in desiccation resistance, starvation resistance and thermal tolerance. It is evident that local adaptation is occurring in *C. capitata* despite high levels of gene flow between populations in South Africa^56^. This may not be surprising considering that repeat introductions often underlie the success of invasive species, with the arrival of new genetic variability providing new traits on which natural selection can act^43^.

Tolerance of dry conditions and stressful temperatures was affected by acclimation, but the response to acclimation was not consistent across *C. capitata* populations or phenotypic trait. Further, acclimation had smaller effects than site on stress tolerance phenotype, which contrasts with results from *D. melanogaster*^55^. Previous studies often assumed that an acclimation response to changing environmental conditions would be beneficial for survival under the new set of conditions. However, this ‘beneficial acclimation hypothesis’ is often not evident, with a range of alternative responses to acclimation being observed^57^. Changes in the CT_min_ and CT_max_ of *C. capitata* in response to a 5-day acclimation treatment tended to follow the pattern predicted by the beneficial acclimation hypothesis, with flies acclimated at cooler temperatures having lower critical thermal limits than those acclimated at higher temperatures. These results coincide with those from previous studies on the thermal tolerance of *C. capitata* sampled from a laboratory culture^31,33,35^, with the magnitude of acclimation response slightly lower for CT_max_, which is in agreement with findings from a range of other insect taxa^58,59^. They also correspond with data showing that acclimation to warmer temperatures facilitates the establishment of invasive species through wider performance breadth^13,22^. However, this was not the case for desiccation resistance and starvation resistance. Desiccation resistance increased with increasing acclimation temperature in *C. capitata* populations from Levubu and Koekoeb, but declined in flies from Ceres and Nuy, 20- and 30 °C-acclimated flies from Wellington and Burgershall performed better than those maintained at 25 °C, and little or no difference in desiccation resistance between acclimation temperatures was evident in populations from Porterville and Nairobi. Starvation resistance in response to increasing acclimation temperature either increased or decreased depending on which population was tested, but none of these effects were significant. This result is in partial agreement with those reported for several widespread Australian *Drosophila* species where there was no evidence for geographic variation in acclimation of desiccation resistance^48^.

The difference in the direction of change of desiccation and starvation resistance, and critical thermal limits of *C. capitata* in response to acclimation temperature may represent the relative value of temperature change as a cue for physiological adaptation in populations living in different bioclimatic regions. For example, while our study found that thermal acclimation had no consistent effects on desiccation and starvation resistance, acclimation in response to a brief period of water stress may have led to very different results^48^. Alternatively, these results may represent the way in which temperature affects underlying physiological processes that then shape tolerance phenotypes. Body size^60,61^, body water^62,63^ and lipid content^64,65^ have been shown to correlate positively with desiccation and starvation resistance in some insect models. All three variables have also been shown to correlate with improved desiccation and starvation resistance in laboratory-reared *C. capitata*, where lipids represent a source of stored energy that can be catabolised to survive periods of food shortage and release metabolic water^41^. In our study, body water content of *C. capitata* tended to decline with increasing acclimation temperature (although again this pattern was not consistent between populations), which may have contributed to the decline in desiccation resistance with increasing acclimation temperature that was noted in some populations. While body lipid and starvation resistance does not seem to be related at the population level, there is a tendency within a population for starvation resistance to be highest in acclimation treatments with higher lipid content. Body lipid content of *C. capitata* tended to be lowest in individuals acclimated at 20 °C, which may indicate that this temperature is suboptimal for nutrient absorption or assimilation. The allocation of nutrients from the diet to different physiological processes at different ambient temperatures has been demonstrated in *Locusta migratoria*^66^, but is yet to be studied in tephritid species.

There was a weak but significant (or marginally so) negative relationship between desiccation and starvation resistance of the tested populations with growing degree-days. Temperature and rainfall-related clines in desiccation and starvation resistance have been identified in *Drosophila* species^50,63,67,68^. Desiccation resistance of *D. busckii* and *D. melanogaster* populations in the western Himalayas increases with altitude, with higher altitudes representing cooler and drier environments^63^. In *D. buzzattii*, desiccation resistance declined and starvation resistance increased as altitude increased^67^, which was presumed to reflect elevational declines in temperature with associated reduction in evaporative water loss and feeding resources^67^. In the case of *C. capitata*, a decline in desiccation and starvation resistance with growing degree-days also suggests a role of resource availability. Adult tephritid fruit flies have been noted to feed on juices from damaged fruit, leaf exudates, bacteria, honeydew (anal secretions) from sap-sucking Hemiptera, pollen, and vertebrate droppings^69–71^, and these sources of nutrients and pre-formed water would be more likely in climates that are favourable for plant productivity. Therefore, while there was no clear relation between body lipid content or desiccation or starvation resistance of adult *C. capitata* in this study, this relationship with growing degree days suggests a need to establish how diet influences these traits.

Population-level patterns of thermal tolerance were significantly associated with variability in temperature seasonality. Although these relationships were weak, as CT_min_ increased with temperature seasonality, there was a corresponding decline in the CT_max_ of *C. capitata* populations, meaning that the widest tolerance breadth was recorded from sites with weakest seasonal fluctuations in temperature. The thermal tolerance of arthropod species has been shown to vary along latitudinal and altitudinal clines associated with changes in temperature and rainfall^51,60,72^. These clinal differences in thermal tolerance have been explained by direct selection for altered survival of temperature extremes^67,72^. Results for *C. capitata* suggest survival of temperature extremes is also important in determining thermal tolerance traits. In *C. capitata*, the change in CT_max_ was less than the change in CT_min_ found by the meta-analyses of Addo-Bediako, *et al*.^4^ and Sørensen, *et al*.^73^. However, Addo-Bediako, *et al*.^4^ found that tolerance breadth should increase rather than narrow in regions with higher variability in temperature. Sites with the highest levels of temperature seasonality in our study were from the Western Cape province of South Africa, which experiences a hot dry summer and cold wet winter^35^. Under these circumstances, it may be that *C. capitata* use behavioural adaptations, such as seeking sheltered microclimates, rather than physiological ones to maintain their body temperature within functional limits.

In conclusion, the results of our study suggest that the invasive potential of *C. capitata* may be supported by adaptation of desiccation and starvation resistance, and thermal tolerance traits to local bioclimatic conditions among populations, despite high gene flow detected from microsatellite markers. With regard to both water and thermal relations, the magnitude of phenotypic plasticity is far less than that attributable to differences between populations. Together, these results suggest that *C. capitata* has a high potential for evolutionary responses to environmental conditions as well as high levels of baseline resistance, which may play a considerable role in its high invasive potential.

## Methods

### Populations

*Ceratitis capitata* were collected from eight sites (Table 1) as larvae in infested fruit and reared to the adult life stage. Sampling time varied depending on host fruit phenology and access to sampling sites (Levubu: coffee, February 2011; Porterville and Wellington: guavas, March 2011; Ceres and Nuy: plums and grapes, April 2011; Koekoeb: grapes, February 2012; Burgershall: coffee, December 2012; Nairobi: January 2013). All sampled populations were within what is considered the native range of *C. capitata*. Fruit were sampled from additional sites in South Africa and Tanzania, but no *C. capitata* were recovered. On adult emergence, 150 adults of mixed sex from each population were transferred to three plastic cages (volume 5 L) furnished with sugar and yeast extract powder (Biolab, Merck, Wadeville, Gauteng, South Africa) in separate dishes for food, water-soaked cotton wool, and a mesh-covered jar with 100 mL of saturated NaCl solution to maintain relative humidity at 75%. All cages were then inserted into clear plastic bags that were sealed and placed in an incubator maintained at 25 °C.

In cases where fewer than 100 adults emerged from field-collected fruit (Burgershall, Koekoeb, Nairobi, Nuy), the adults were kept to establish a laboratory culture so that sufficient numbers for experiments could be reared. Adults sourced from field-collected fruit were transferred to large polycarbonate cages in a constant environment room (25 ± 1 °C, 65% relative humidity, 12:12 photocycle). In addition to food and water, as described above, adults were provided with bananas into which females could oviposit. To aid oviposition and egg survival the peels of the bananas were pierced with numerous holes using a needle. Bananas were removed from the adult cage and replaced twice each week. Potentially infested bananas were transferred to a ventilated container that was lined with a layer of fine vermiculite that wandering third instar larvae could burrow into to pupate. The vermiculite was gently sieved from the third day after incubation and successively every day for four days to obtain pupae, which were placed in a new cage to emerge. Significant changes in the age of mating and attainment of peak egg load can occur in as few as four generations^74^, so cultures from each site were kept for no more than four generations before testing to minimise adaptation to the laboratory environment. In some instances, this condition reduced the number of individuals available for experiments.

### Acclimation

Changes in tolerance resulting from acclimation to controlled temperature manipulation in the laboratory were regarded as representing phenotypic plasticity. At 5 days after adult emergence one cage holding flies from each site was transferred from 25 °C to 20 °C. Another cage was transferred to an incubator held at 30 °C. The remaining cage was left at 25 °C. The flies were held under these new conditions for 5 days, which has been demonstrated to provide ample time for *C. capitata* to exhibit temperature-related changes in phenotype^34^. Temperature and relative humidity in each cage was verified using data loggers (iButton DS1923, Maxim, Sunnyvale, CA, USA).

### Initial condition

At 10 days after adult emergence, up to 10 flies (ideally 5 females and 5 males, numbers vary according to mortality) from each population and acclimation treatment were placed in microcentrifuge tubes of known weight. The initial mass of each fly was measured (to 0.0001 g) on an analytical balance (MS104S, Mettler Toledo, Switzerland) before being stored in a freezer at −20 °C. At a later stage, the flies were placed in a fan-forced drying oven at 60 °C for 96 hours. Dry mass was measured (to 0.000001 g) on a microbalance (UMX2, Mettler Toledo, Switzerland) after allowing flies to cool to laboratory temperature for 15 minutes. Dry mass (rounded to 0.0001 g) was subtracted from initial mass to estimate initial body water content.

Dried flies were then soaked in 0.5 mL of choroform:methanol (1:1) solution for 1 hour to extract body lipids^75,76^. The solution was decanted and the flies permitted to air-dry overnight. This was repeated a further two times. Flies were then placed in the fan-forced drying oven at 60 °C for another 96 hours before their lipid-free dry mass was measured (to 0.000001 g). Lipid-free dry mass was subtracted from dry mass to determine initial body lipid content.

### Desiccation and starvation resistance

Desiccation and starvation resistance were determined following methods modified from Gibbs, *et al*.^62^. At 10 days after adult emergence, 20 females and 20 males from each site and acclimation treatment (120 flies per population) were placed into individual, numbered, pre-weighed 2 mL microcentrifuge tubes pierced with 12 holes (approximate diameter = 1 mm). Flies in tubes were then weighed (to 0.0001 g) and initial mass of each fly was calculated by subtracting tube mass. Of these tubes, 10 females and 10 males from each site and acclimation treatment were then transferred to three desiccators containing silica gel (desiccation resistance assay, 0–10% relative humidity), whereas the others were transferred to three desiccators containing distilled water (starvation resistance assay, 90–100% relative humidity). Maintaining flies at close to 100% relatively humidity acts as a starvation treatment because flies are able to imbibe water that condenses on the inner surface of the tube (due to small fluctuations in incubator temperature to below dew point). Desiccators used for the desiccation and starvation resistance assays were labelled A, B or C. Sites, sexes and acclimation treatments were haphazardly distributed within the three desiccators. Each tube was inverted and secured to the plate of the desiccator with a small ball of putty-like temporary adhesive (Prestik, Bostik, Permoseal (Pty) Ltd, Chempet, South Africa). All desiccators were then placed in an incubator maintained at 25 °C. Temperature and relative humidity data loggers were used to measure conditions in each desiccator.

All tubes were checked for mortality every 3 hours by viewing them through the clear plastic top of the desiccators. Dead flies were removed from their tubes, weighed (to 0.0001 g), returned to their tubes and stored in a freezer at −20 °C.

After all flies had died they were placed in a fan-forced drying oven at 60 °C for 96 hours. Dry mass was measured (to 0.000001 g) after allowing flies to cool to laboratory temperature for 15 minutes. Dry mass (rounded to 0.0001 g) was subtracted from body mass at death to estimate water remaining at death. Methods described above to extract body lipids were followed to determine lipid content remaining at death.

### Critical thermal limits

Ten individual *C. capitata* from each site and acclimation were placed into a double jacketed chamber (‘organ pipes’) connected to a programmable circulating refrigerated bath (Huber CC 410 WL, Offenburg, Germany) filled with 1:1 water: propylene glycol to allow for subzero temperatures^32^. This process was repeated twice to yield sample sizes of n = 20 flies per treatment where sufficient flies were available. The order in which flies from each acclimation treatment were tested was randomised to avoid any potential diel effects on thermal limits. A thermocouple (type T, 36 SWG) connected to a digital thermometer (Fluke 54 series II, Fluke Cooperation, China; accuracy: 0.05 °C) was inserted into the middle/control chamber to record chamber temperature. For all critical thermal limit experiments, we assumed that body temperature of adult *C. capitata* individuals was always in equilibrium with chamber temperature under the experimental conditions employed, as has been demonstrated for other insects^77,78^. Both critical thermal maximum (CT_max_) and critical thermal minimum (CT_min_) experiments started at a setpoint temperature of 25 °C (with 10 minutes equilibration time) from which temperature increased for CT_max_ or decreased for CT_min_ at a rate of 0.25 °C/min until all the flies reached their CT_max_/CT_min_. This ramping rate was chosen to maximise throughput of individuals per day, but also being relatively ecologically-relevant compared to much of the work undertaken on critical thermal limits to date (see discussion in Chown *et al*.^79^). Critical thermal limits were defined as the temperature at which each individual insect lost co-ordinated muscle function^80^, consequently losing the ability to respond to mild stimuli (e.g. prodding). In the case of CT_max_, this loss of muscle function coincided with death such that recovery was not possible, while in the case of CT_min_, recovery occurred, and hence, was not immediately lethal.

### Data analysis

#### Initial condition

Initial body mass, dry mass, water content and lipid content were analysed with nested analysis of variance (ANOVA) using the general linear model dialogue of Statistica 12 (StatSoft). For initial body mass, the effect of acclimation was nested within the effect of site. The same effects, plus that of initial body mass as a covariate, were included in the models for initial dry mass, body water content and lipid content. The percentage of variance accounted for by each effect was calculated for this and all following linear models following Sokal and Rohlf^81^. Post-hoc pair-wise least significant difference tests were used to identify homogenous subsets within each nested level. In these analyses, as well as all subsequent ones, it was decided that sex would not be included as an effect in statistical models because this study aimed to assess overall population-level patterns. More detailed analysis of sex differences in thermal tolerance have been published elsewhere^32^, and those for desiccation resistance are reported in Weldon, *et al*.^41^.

#### Desiccation and starvation resistance, and thermal tolerance traits

Data from the desiccation and starvation resistance assays were explored using parametric survival analyses with Weibull hazard function in R Studio version 0.99.902 running R version 3.3.1^82^. Desiccation and starvation results were analysed separately. In both cases, the model for time to death included effects of site, acclimation nested within site, and body mass as a covariate.

Dehydration tolerance, defined as body water remaining at death when corrected for initial body water^62^, was determined for *C. capitata* from each site and acclimation treatment. To do so, initial body water was predicted based on initial body mass of desiccated flies using regression equations of initial body water content on initial body mass from control flies (Table S2). Linear modelling was performed in Statistica to assess the effects of site, acclimation nested within site, and estimated body water content on body water remaining at death of desiccated flies. Post-hoc pair-wise least significant difference tests were used to identify homogenous subsets within each nested level.

Lipid content remaining at death was natural log-transformed before being analysed using a linear model. The effects of assay (i.e., desiccation or starvation resistance), nested within acclimation, and nested within site were included in the model, as well as initial mass and survival time as covariates. In this case, initial lipid content was not estimated and included in the model because while there was an overall significant effect of body mass on initial lipid content (Table S1) the relationship with initial body mass was very weak. Homogenous subsets were identified with post-hoc pair-wise least significant difference tests.

CT_max_ and CT_min_ were analysed using nested ANOVA in Statistica. The effects of site nested within acclimation were included in the model. Post-hoc pair-wise least significant difference tests were used to identify homogenous subsets.

#### Association with bioclimatic variables and population relatedness

Bioclimatic characteristics of each site were extracted from Metzger, *et al*.^44^ (Table 1). The characteristics targeted were the four bioclimatic characteristics identified by Metzger, *et al*.^44^ as the most important for constructing their global environmental stratification (GEnS): potential evapotranspiration seasonality [PET (sd); standard deviation of monthly mean potential evaporation × 100], growing degree-days with 0 °C base (GDD, calculated on monthly temperature means above 0 °C × number of days in the month), aridity index (mean annual precipitation divided by mean annual evapotranspiration), and temperature seasonality [Temp (sd); standard deviation of monthly mean temperature × 100]. These four variables alone account for 99.9% of global environmental variability^44^, so are more informative for describing the selective environment experienced by different *C. capitata* populations throughout their range than other bioclimatic characteristics such as average temperatures or rainfall [which are highly correlated with PET, GDD, aridity index, and Temp (sd)^44^].

Mean values for desiccation and starvation resistance, lipid content, and CT_max_ and CT_min_ at 25 °C, as well as the acclimation response difference for each of these variables, were related to all bioclimatic characteristics using ordinary least-squares regression in R. The acclimation response difference was the trait mean at 30 °C minus the trait mean at 20 °C, and is akin to the “acclimation response ratio” proposed by Claussen^83^. The minimal adequate model was determined by step-wise deletion of the least significant terms using the ‘step’ command to minimise Akaike’s information criterion (AIC) and the number of degrees of freedom in the model.

The influence of among population genetic structure of the sampled *C. capitata* populations on trait means and acclimation response differences was also determined. Microsatellite data matching sampling locations in this study were obtained from two earlier studies^26,56^ to use in phylogenetic tree construction. Individuals included were rescored in GENEMAPPER v3.7 (Applied Biosystems). Using POPTREEW^84^ an unrooted neighbour-joining tree was constructed based on Nei’s genetic distance (DA^85^) and statistical support for the branches were assessed using non-parametric bootstrapping (10,000 replicates) (Fig. 1A). The resulting phylogeny was imported into R as a tree file. The resulting phylogenetic tree was unresolved with relatively low bootstrap values, but this is not surprising as there is no population genetic structure between *C. capitata* populations within South Africa^56^, or even between populations in Africa^26^. Using the ‘caper’ library, the phylogeny (imported as a tree file) was matched with the trait means and acclimation response differences for each population using the ‘comparative.data’ command. Phylogentic least squares regression using the function ‘pgls’ was then used to determine whether significant effects identified with the linear minimal adequate models remained so when accounting for evolutionary relationships among populations. A maximum likelihood approach was used to establish whether trait evolution was phylogenetically correlated (by determining λ^86^). The fit of the minimal adequate ordinary least-squares regression and the phylogenetically corrected model to the data for each variable was assessed using their AIC values.

## Electronic supplementary material

Supplementary information
