# Supplementary material for: Geographic variation and plasticity in climate stress resistance among southern African populations of Ceratitis capitata (Wiedemann) (Diptera: Tephritidae)
Source: Sci Rep. 2018 Jun 29;8:9849. doi: 10.1038/s41598-018-28259-3 (PMC6026165; doi:10.1038/s41598-018-28259-3)
Supplement: Supplementary file 1 — Supplementary information [file 41598_2018_28259_MOESM1_ESM.pdf]

SUPPLEMENTARY INFORMATION

Geographic variation and plasticity in climate stress resistance among southern African populations of *Ceratitis capitata* (Wiedemann) (Diptera: Tephritidae)

Christopher W. Weldon[1,*], Casper Nyamukondiwa[2†], Minette Karsten[2], Steven L. Chown[3] and John S. Terblanche[2]

[1] Flies of Economic Importance Research Group, Department of Zoology and Entomology, University of Pretoria, Private Bag X20, Hatfield 0028, South Africa

[2] DST-NRF Centre for Invasion Biology, Department of Conservation Ecology and Entomology, Stellenbosch University, Private Bag X1, Matieland 7602, South Africa

[3] School of Biological Sciences, Monash University, Victoria 3800, Australia

†Current address: Department of Biological Sciences and Biotechnology, Botswana International University of Science and Technology (BIUST), Private Bag 16, Palapye, Botswana

* Author for correspondence: cwweldon@zoology.up.ac.za

Table S1. Summary of the linear models that describe the effects of acclimation nested within site on the initial mass, body water content, dry mass and body lipid content of *C. capitata* (n = 419). Percent variance was calculated as eta squared (η^2, also known as the correlation ratio or R^2 when df=1).

Effect	SS	df	MS	*F*	*P*	% variance
Initial mass						
Intercept	13520.35	1	13520.35	7408.41	0.001	95.4
Site	243.98	7	34.85	19.098	**<0.001**	1.7
Acclimation	40.01	16	2.5	1.37	0.159	0.3
Error	361.35	198	1.82			2.6
Body water content						
Intercept	1.72	1	1.72	18.75	<0.001	1.2
Site	13.26	7	1.89	20.70	**<0.001**	9.3
Acclimation	4.94	16	0.31	3.38	**<0.001**	3.5
Initial mass	104.67	1	104.67	1143.97	**<0.001**	73.4
Error	18.03	197	0.09			12.6
Dry mass						
Intercept	1.68	1	1.68	18.61	<0.001	1.5
Site	13.00	7	1.86	20.57	**<0.001**	11.4
Acclimation	4.85	16	0.30	3.35	**<0.001**	4.2
Initial mass	76.74	1	76.74	849.97	**<0.001**	67.3
Error	17.79	197	0.09			15.6
Body lipid content						
Intercept	0.12	1	0.12	2.37	0.126	0.4
Site	12.11	7	1.73	34.12	**<0.001**	44.7
Acclimation	1.98	16	0.12	2.45	**0.002**	7.3
Initial mass	2.90	1	2.90	57.15	**<0.001**	10.7
Error	9.99	197	0.05			36.9

Table S2. Linear regression for the relationship between body mass (mg) and body water content (mg) for eight populations of *Ceratitis capitata* that were subsequently tested for desiccation resistance and water loss rate. Females and males from each populations were acclimated for five days to constant temperatures of either 20, 25 or 30˚C before water balance assays were performed. The equation for each relationship was used to estimate initial body water content from initial body mass for flies subjected to desiccation assays. Sexes were pooled for acclimation temperatures where fewer than five individuals of each sex were available to sample.

Cohort	Equation	R^2	F	df	P-value
Nuy					
20˚C					
Female	y = -2.07 + 0.87x	0.636	5.249	1, 3	0.106
Male	y = -0.33 + 0.69x	0.861	18.646	1, 3	0.023
25˚C					
Both sexes	y = 0.99 + 0.48x	0.835	40.548	1, 8	<0.001
30˚C					
Female	y = -0.26 + 0.65x	0.949	55.278	1, 3	0.005
Male	y = 0.69 + 0.57x	0.989	268.810	1, 3	<0.001
Wellington					
20˚C					
Female	y = 2.22 + 0.38x	0.969	62.376	1, 2	0.016
Male	y = 1.53 + 0.43x	0.860	24.473	1, 4	0.008
25˚C					
Female	y = 1.19 + 0.45x	0.879	21.880	1, 3	0.018
Male	y = 0.81 + 0.47x	0.922	35.434	1, 3	0.009
30˚C					
Female	y = -0.06 + 0.56x	0.988	161.708	1, 2	0.006
Male	y = 1.23 + 0.41x	0.882	29.904	1, 4	0.005
Porterville					
20˚C					
Female	y = 0.81 + 0.51x	0.542	3.547	1, 3	0.156
Male	y = -0.10 + 0.63x	0.922	35.354	1, 3	0.010
25˚C					
Female	y = 2.83 + 0.28x	0.382	1.853	1, 3	0.267
Male	y = 0.28 + 0.54x	0.925	37.072	1, 3	0.009
30˚C					
Female	y = 0.54 + 0.50x	0.549	3.647	1, 3	0.152
Male	y = 0.70 + 0.46x	0.911	42.062	1, 3	0.007
Ceres					
20˚C					
Both sexes	y = 1.39 + 0.46x	0.900	26.965	1, 3	0.014
25˚C					
Both sexes	y = 0.67 + 0.51x	0.594	7.313	1, 5	0.043
30˚C					
Both sexes	y = 0.37 + 0.51x	0.909	60.033	1, 6	<0.001

Table S2. Continued.

Cohort	Equation	R^2	F	df	P-value
Burgershall					
20°C					
Female	y = 0.80 + 0.56x	0.995	653.399	1, 3	<0.001
Male	y = -0.13 + 0.65x	0.890	24.188	1, 3	0.016
25°C					
Female	y = 1.84 + 0.43x	0.878	21.564	1, 3	0.019
Male	y = 1.38 + 0.44x	0.764	9.691	1, 3	0.053
30°C					
Female	y = 0.39 + 0.60x	0.956	65.539	1, 3	0.004
Male	y = -0.95 + 0.80x	0.981	156.090	1, 3	0.001
Koekoeb					
20°C					
Female	y = -0.71 + 0.71x	0.941	47.584	1, 3	0.006
Male	y = 0.78 + 0.51x	0.829	14.513	1, 3	0.032
25°C					
Both sexes	y = -0.99 + 0.77x	0.943	65.944	1, 4	0.001
30°C					
Female	y = 0.37 + 0.55x	0.962	76.384	1, 3	0.003
Male	y = -0.24 + 0.70x	0.946	52.485	1, 3	0.005
Levubu					
20°C					
Both sexes	y = 0.73 + 0.47x	0.510	8.341	1, 8	0.020
25°C					
Female	y = -0.38 + 0.68x	0.853	23.281	1, 4	0.008
Male	y = 1.22 + 0.38x	0.852	11.478	1, 2	0.077
30°C					
Both sexes	y = -0.34 + 0.58x	0.946	69.866	1, 4	0.001
Nairobi					
20°C					
Both sexes	y = 1.83 + 0.41x	0.566	10.447	1, 8	0.012
25°C					
Both sexes	y = 0.83 + 0.53x	0.821	36.629	1, 8	<0.001
30°C					
Both sexes	y = 1.37 + 0.47x	0.789	29.943	1, 8	0.001

Table S3. Summary of the linear model that describes the effects of acclimation nested within site and estimated total body water on water remaining at death in *C. capitata* that were subjected to a desiccation resistance assay (n = 479). Values for total body water were estimated using the linear regression equations presented in Table S1.

Effect	SS	df	MS	*F*	*P*	% variance
Intercept	53.78	1	53.78	149.62	<0.001	20.2
Site	37.94	7	5.42	15.08	**<0.001**	14.2
Acclimation	8.98	16	0.56	1.56	0.075	3.4
Total body water	2.40	1	2.40	6.68	**0.010**	0.9
Error	163.18	454	0.36			61.3

Table S4. Summary of the linear model that describes the effects of stress resistance assay (desiccation or starvation) nested within acclimation, acclimation nested within site, and initial body mass and survival time on body lipid content remaining at death in *C. capitata*.

Effect	SS	df	MS	*F*	*P*	% variance
Intercept	37.06	1	37.06	157.71	<0.001	10.6
Site	26.33	7	3.76	16.01	**<0.001**	7.5
Acclimation	7.37	14	0.53	2.24	**0.005**	2.1
Assay	55.59	3	18.53	78.85	**<0.001**	15.8
Initial body mass	0.04	1	0.04	0.17	0.676	<0.1
Survival time	6.38	1	6.38	27.16	**<0.001**	1.8
Error	218.08	928	0.24			62.2

Table S5. Coefficients from the minimal adequate ordinary least-squares regression (OLS) and phylogenetic generalised least squares regression (PGLS) models for the acclimation response of desiccation resistance, starvation resistance, lipid content, critical thermal minimum (CT_{min}) and critical thermal maximum (CT_{max}) of *C. capitata* populations. The minimal adequate least squares regression model was determined using step-wise deletion of least significant terms based on improvement of Akaike's information criterion (AIC). Predictor variables in the full model were potential evapotranspiration seasonality [PET (sd)], growing degree-days with 0˚C base (GDD), aridity index, and temperature seasonality [Temp (sd)]. Phylogenetic correlation (on a scale of 0 to 1) is given by λ.

Effect	OLS					PGLS					
	Estimate	S.E.	*t*	*P*	AIC	Estimate	S.E.	*t*	*P*	AIC	λ
Desiccation											
(Intercept)	4.136	11.343	0.365	0.730	63.953	4.709	11.416	0.413	0.697	62.105	0.000
PET	-0.022	0.009	-2.444	0.058		-0.022	0.009	-2.500	0.054		
Temp (sd)	0.027	0.012	2.267	0.073		0.027	0.012	2.310	0.069		
Starvation											
(Intercept)	85.906	55.180	1.557	0.180	71.850	84.896	55.350	1.534	0.186	69.960	0.000
GDD	-0.015	0.007	-2.027	0.099		-0.015	0.007	-1.978	0.105		
Temp (sd)	0.006	0.005	1.217	0.278		0.006	0.005	1.207	0.281		
Lipid content											
(Intercept)	0.107	0.093	1.151	0.288	4.193	0.115	0.093	1.231	0.258	2.360	0.000
CT_{min}											
(Intercept)	0.510	0.337	1.515	0.181	7.153	0.526	0.340	1.548	0.173	5.338	0.000
Temp (sd)	0.000	0.000	1.568	0.168		0.000	0.000	1.527	0.178		
CT_{max}											
(Intercept)	0.772	0.111	6.973	<0.001	7.055	0.786	0.108	7.290	<0.001	4.647	0.000
